# A research protocol for a pilot randomized controlled trial designed to examine the feasibility of a couple-based mind-body intervention for patients with metastatic lung cancer and their partners

**DOI:** 10.1186/s40814-018-0231-6

**Published:** 2018-01-24

**Authors:** Kathrin Milbury, Anne S. Tsao, Zhongxing Liao, April Owns, Rosalinda Engle, Edrea A. Gonzalez, Eduardo Bruera, Lorenzo Cohen

**Affiliations:** 1 Department of Palliative, Rehabilitation and Integrative Medicine, The University of Texas MD Anderson Cancer Center, Unit 1414, 1515 Holcombe Boulevard, Houston, TX 77030 USA; 2 Department of Thoracic/Head and Neck Medical Oncology, The University of Texas MD Anderson Cancer Center, Houston, TX USA; 3 Department of Radiation Oncology, The University of Texas MD Anderson Cancer Center, Houston, TX USA

**Keywords:** Metastatic non-small cell lung cancer, Couples, Mind-body intervention, Feasibility, Randomized control trial, Study protocol, Quality of life

## Abstract

**Background:**

Given the generally incurable nature of metastatic non-small cell lung cancer (mNSCLC), patients and their romantic partners are at risk for existential/spiritual distress. Although a handful of dyadic psychosocial interventions for lung cancer patients and their caregivers exist, none of them target spiritual well-being. Informed by the mindfulness-based intervention literature and our pilot work in couples affected by lung cancer, we developed a brief couple-based mind-body (CBMB) intervention. The primary aim of this research protocol is to determine the feasibility of implementing the CBMB intervention versus an active control (AC) or wait list control (WLC) group in patients with mNSCLC and their partners using a randomized controlled trial design.

**Methods:**

Seventy-five patients with mNSCLC receiving treatment and their partners are randomized to the CBMB intervention, an AC or a WLC group. Those in the CBMB intervention and AC groups receive four intervention sessions of 60 min each over 4 weeks and complete weekly homework assignments. The first session is delivered in person, and the remaining sessions are delivered via videoconference. The dyads in the AC group discuss cancer-related and personal growth concerns with the interventionist but are not taught coping skills. Patients and partners in all groups complete baseline assessments of quality of life (QOL) prior to randomization. Follow-up assessments are performed 4 weeks and then again 3 months later. The primary outcome is feasibility (i.e., ≥ 30% of eligible couples consent, ≥ 70% of enrolled couples are retained, and ≥ 50% of all CBMB and AC sessions are attended). We will also perform primarily descriptive analyses of the self-reported outcomes (e.g., spiritual well-being and psychological distress) and explore potential intervention mediators (i.e., compassion, communication, mindfulness, and closeness) to inform a larger, future trial.

**Discussion:**

This trial will provide important information regarding the feasibility of a behavioral intervention in a vulnerable yet understudied population using videoconferencing and descriptive data regarding spiritual well-being and other indices of QOL in both mNSCLC patients and their partners.

**Trial registration:**

ClinicalTrials.gov NCT02596490

## Background

Given the high symptom burden and generally incurable nature of metastatic non-small cell lung cancer (mNSCLC), spiritual distress (e.g., lack of meaning and hopelessness) is common in patients and their families [1]. Although patients with advanced cancer tend to express spiritual needs and desire spiritual care, their spiritual and/or existential concerns and needs remain largely unmet [2]. For instance, in a recent study, 44% of patients with advanced cancer reported spiritual distress defined by the presence of at least two of seven domains (despair, dread, brokenness, helplessness, alienation, meaninglessness, and shame/guilt), with despair being the most frequent spiritual distress domain [3]. Spiritual pain (described as “pain deep in the soul or being that is not physical”), endorsed by 44% of patients with advanced cancer, is adversely related to physical and emotional symptoms [1]. In contrast, spiritual well-being (sense of meaning, faith, and peace) protects against the desire for a hastened death, hopelessness, and suicidal ideation *independent* of depression, social support, physical function, and cancer symptoms in patients with terminal cancer [4]. Thus, spiritual well-being is an important aspect of QOL management in this patient population.

Because of mNSCLC patients’ limited life expectancy, it is not surprising that their family members, particularly spouses and partners, are also vulnerable to spiritual pain (58% in one sample) [5]. Caregivers with high levels of spiritual pain report higher levels of anxiety, depression, denial, and other dysfunctional coping strategies than do those who do not report spiritual pain [6]. As partners cope with their own fears and spiritual/existential distress, providing emotional support to patients may be difficult [7]. Also, partners’ spiritual well-being is associated with their own as well as patients’ QOL [8].

Considering that QOL, including spiritual dimensions, is interdependent in couples coping with cancer (patient outcomes influence partner outcomes and vice versa), representing a relational system, a dyadic intervention may optimize the efficacy of the treatment response [8]. Although the majority of dyadic psychosocial intervention research has focused on couples coping with localized breast or prostate cancer, the literature points to a handful of randomized controlled trials (RCTs) for couples dealing with lung cancer [9]. However, few of them included patients with metastatic disease, none of the interventions were designed to address existential and/or spiritual concerns, and they did not target spiritual well-being as the primary outcome.

### Objectives

To address the gaps in the literature regarding dyadic psychosocial interventions for lung cancer patients and their romantic partners, we developed a mindfulness-based intervention designed to target the psychospiritual needs of couples affected by mNSCLC. Informed by the mindfulness-based intervention literature for cancer and our previous work suggesting that a meditation program increases spiritual well-being and reduces distress outcomes in patients with stage I–III lung cancer and their partners [10, 11], we developed a brief couple-based mind-body (CBMB) intervention. Because couples coping with cancer frequently report social constraints to open communication regarding cancer-related concerns, we integrated partner-assisted emotional disclosure, a technique that has been shown to improve cancer adjustment [12]. Thus, the main components of our CBMB program (mindfulness and emotional disclosure) may work synergistically to facilitate cancer adjustment via intrapersonal and interpersonal connection. We now seek to collect data on the feasibility of implementing a pilot RCT of our CBMB intervention, including the administration of self-reported QOL measures, for mNSCLC patients and their partners. Additionally, we will obtain descriptive evidence of self-reported outcomes to inform a subsequent clinical trial.

In summary, our specific aims are as follows:

1. To determine the feasibility of performing an RCT for patients with mNSCLC and their partners involving the CBMB intervention, active control (AC), and usual care wait list control (WLC) groups.
2. To perform descriptive analyses of QOL outcome measures in patients and their partners.

As an exploratory aim, we will carry out descriptive analyses including correlations between QOL measures and measures of potential mediators (i.e., mindfulness, compassion, holding back, and intimacy) to help provide the basis for an underlying mechanism of the intervention benefits to be further explored in a future larger trial.

## Methods

### Design overview and study setting

An RCT is currently conducted with 75 dyads randomized to the intervention, AC, or WLC group. The couples complete the initial QOL assessments prior to randomization. Follow-up assessments are completed within 1 week of completing the intervention and again 3 months later. For each completed questionnaire, participants receive a $20 gift card ($40 per couple). Participants’ parking costs for in-person appointments is paid if they are not covered by the hospital. Couples in the CBMB intervention group attend four weekly sessions (60 min each) over 4 weeks. Couples in the AC group discuss cancer and relationship-related topics in a structured and factual manner to control for possible effects of time and attention (four total sessions over 4 weeks, 60 min each). Couples in the WLC group receive usual care.

### Eligibility

#### Inclusion criteria

Patients must (1) be diagnosed with mNSCLC, (2) be receiving treatment (e.g., radiotherapy and chemotherapy) at MD Anderson Cancer Center, (3) have an Eastern Cooperative Oncology Group (ECOG) performance score no higher than 2, and (4) have a romantic partner with whom they have resided for a minimum of 6 months. Patients and partners must be (1) at least 18 years old, (2) able to read and speak English, and (3) able to provide informed consent.

#### Exclusion criteria

Patients and partners (1) not oriented to time, place, or person as deemed by the clinical team and (2) regularly (self-defined) participate in psychotherapy or a formal cancer support group will be excluded from participation.

### Interventions

#### CBMB intervention group

The meditation component of the CBMB intervention is based on our completed pilot work in meditation [13, 14]. We use mindfulness-based breathing exercises and compassion-based meditations such as lovingkindness mediations as a basis for our intervention. Researchers have extensively studied both mindfulness-based breathing and lovingkindness meditations and deemed them effective stress-reduction techniques [10, 15–21]. An overview of each CBMB intervention session is presented below. Session 1 is completed face-to-face or online via videoconferences depending on the participants’ availability. Sessions 2–4 are delivered via videoconferencing. A master’s level mind-body intervention specialist who is experienced in working with cancer patients and their families will implement the sessions.

#### Session 1

The first session starts with a brief introduction and overview of the program. The session focuses on providing instructions regarding mindfulness meditation techniques. The interventionist introduces couples to appropriate meditation postures, breathing awareness, concentration or focused attention, and mindfulness using simple visualizations. These initial practices help couples learn concentration/focused attention and mindfulness in the sitting posture. Couples are taught the three aspects of mindfulness: being in the present moment, not being judgmental, and being intentional. Couples share their experiences regarding these exercises, and the interventionist explains the homework assignment and procedures regarding online accessibility of the subsequent sessions and booster telephone calls.

#### Session 2

The session starts with a brief relaxation exercise. Then, the instructor reviews the homework assignment from the previous session. In session 2, couples are introduced to the idea of compassion and creating positive emotions. This session includes techniques such as guided visualizations and a lovingkindness meditation that are intended to facilitate connections between the partners. Couples also learn the ground rules of mindful and compassionate listening and sharing, as they are asked to engage in an emotional disclosure task. Homework is assigned.

#### Session 3

The session starts with a brief relaxation exercise. Then, the instructor reviews the homework assignment from the previous session. During session 3, couples are taught to reflect on things, events, and people they are grateful for via a gratitude meditation. Couples share their experiences of practicing gratitude. The interventionist reminds the couple of the ground rules for sharing prior to the emotional disclosure exercise and facilitates the conversation, if necessary, during sharing exercise. The patient and partner take turns sharing with and listening to each other. Homework is assigned.

#### Session 4

The session starts with a brief relaxation exercise. Then, the instructor reviews the homework assignment from the previous session. In session 4, couples learn strategies to live according to their values. They first engage in a reflection exercise and then complete a worksheet to help identify their most important values. Couples brainstorm to formulate strategies to ensure that their lives reflect their self-identified values. The interventionist reviews all of the tools the couples learned about over the course of the program and helps them proactively identify strategies to continue to implement the tools in their daily lives. Couples also receive instructions on how to continue with this program when no longer meeting with the interventionist.

Couples attend the sessions one-on-one with the instructor. They are encouraged to provide oral feedback regarding the intervention content during class and asked to complete a detailed written evaluation of each component of the intervention including any comments to improve the program. Couples receive compact discs and printed materials with instructions regarding home practice exercises at session 1 and are encouraged to practice the meditation component daily together or individually and disclose their reflections to each other. The interventionist makes at least one booster telephone call per week over the 4-week intervention period. The phone call is intended as a homework reminder and addresses any questions regarding the homework.

Throughout the sessions, the interventionist ensures that couples can successfully implement the meditation component and other exercises learned in class on their own and in the context of everyday situations to help manage symptoms that may arise (e.g., focus on their body to notice how they are feeling, breathing exercises to reduce anxiety and increase awareness, calming their mind to manage sleep disturbances, and mindful listening when the partner shares concerns). At each session and during the booster calls, couples will be asked to identify barriers to implementing the skills and develop strategies to remove barriers (if any) to successfully performing the exercises daily.

#### AC group

Dyads in the AC group complete four sessions (60 min each) over 4 weeks led by a master’s level mind-body intervention specialist. The scheduling of the sessions is identical to that for the CBMB intervention group. The first session is either face to face or online. Sessions 2–4 are delivered via videoconference. The AC sessions are modeled after the social support intervention used by Breitbart and colleagues [22]. However, the original protocol is modified to ascertain an appropriate condition for comparison with the current intervention to be examined. Specifically, AC sessions do not occur in a group setting. Instead, identical to the CBMB intervention group, couples attend one-on-one sessions with the interventionist. The sessions focus on discussing cancer-related concerns that couples tend to experience when coping with metastatic cancer, such as communicating with health care providers; coping with family and friends; caregiving issues; sexual intimacy concerns; relationship changes; coping with physical functioning concerns; fears about future physical or psychological changes, recurrence, and mortality; and future plans. Using a reflective listening approach, the interventionist focuses on encouraging patients and partners to share their concerns with each other. The interventionist does not offer education, support (other than reflective listening), or any other therapeutic tools to the participants. Additionally, the interventionist does not probe for deep emotional disclosure. A comparison group that discusses cancer-related concerns is viewed as credible by patients and matches time and attention as well as nonspecific treatment effects such as those provided by social interactions. Patients’ willingness to participate in this type of comparison group is evidenced by findings of previous studies indicating that attendance of these types of sessions is comparable with that of other intervention groups [22–24] and that credibility ratings are high (8–9 on a 10-point scale) [23]. Couples in the AC group also complete a detailed written evaluation of the AC content.

#### WLC group

Participants in the WLC group receive usual care as provided by the MD Anderson health care team for cancer patients and complete all assessments during the same time frame as the active groups except for the intervention evaluations. After the last assessment, they are offered the CBMB intervention. If couples are interested and chose to participate, no additional questionnaire data are collected.

#### Interventionist training and quality control

The interventionists have experience in working with cancer patients, receive an instructor’s version of the CBMB and AC manuals including scripts and scenarios, and will have role-played with members of the research team before implementing the CBMB intervention and AC programs. To ensure treatment fidelity, all sessions for both active groups are audio recorded with the participants’ permission (obtained during the informed consent process). Using a fidelity checklist, all audio recordings are reviewed by a researcher (KM) to ascertain whether each intervention component was appropriately delivered and whether any extraneous material was included. The sessions are reviewed on an ongoing basis so that feedback can be provided to the interventionists as necessary.

### Study measures

#### Demographic and medical factors

Both patients and partners are asked for demographic information including age, sex, race/ethnicity, marital status/length, occupational status, and educational history. Patient information regarding treatment information, comorbidities, and other background information (e.g., current/past nicotine use) are obtained from medical records.

#### Feasibility

Consent *rates* including refusal reasons, study attrition, class attendance, and completion of each questionnaire and homework assignment (for participants in the CBMB intervention group) are documented. At each session, participants are asked to evaluate each aspects of the program, report any difficulties they may have experienced, communicate whether they perceive any benefit from the CBMB intervention program, and rate the interventionist.

#### QOL measures

Study staff involved in data collection are blinded to the treatment conditions. Self-reported measures were chosen based on their demonstrated psychometric properties, intervention targets, relevance to the targeted population, and brevity. Both patients and partners in all groups complete self-reported measures assessing:

1. *Spirituality* using the Functional Assessment of Cancer Therapy-Spiritual Well-Being Scale (FACT-SP), a 12-item instrument assessing the spiritual dimensions of peace, faith, and meaning [25].
2. *Psychological adjustment* using the Center for Epidemiologic Studies Depression Scale [26], a 20-item self-reported measure focusing on the affective component of depression, and the Impact of Events Scale, a 15-item scale assessing intrusive thoughts and avoidance behaviors [27].
3. *Sleep disturbances* using the Pittsburgh Sleep Quality Index [28], an 18-item self-rated questionnaire that assesses quality of sleep and sleep disturbances.
4. *QOL* using the MD Anderson Symptom Inventory-Lung Cancer module, which consists of 13 core items and 3 lung cancer-specific items assessing symptom severity and interference with daily life (patients only) [29], and the Medical Outcomes Study, a 36-item short-form survey assessing eight distinct domains of mental and physical health (partners only) [30].

#### Potential intervention process measures (completed at the end of treatment and 3 months later)

To explore associations of QOL measures with intervention targets and work toward identifying an underlying mechanism of the CBMB intervention efficacy to be tested in future research, we administer self-reported instruments including the following:

1. Mindful Attention Awareness Scale, a 15-item scale designed to assess a core characteristic of dispositional mindfulness, namely, open or receptive awareness of and attention to what is taking place in the present [31].
2. Self-Compassion Scale, a 12-item questionnaire that provides questions about self-judgments, self-kindness, common humanity, isolation, mindfulness, and overidentification [32].
3. Holding back will be examined using an adapted version of Pistrang’s and Barker’s scale [33] assessing 13 common cancer-related concerns (e.g., emotional problems and financial issues) and the degree to which couples discuss them with each other [12, 34–36].
4. Relationship functioning will be assessed using the six-item Personal Assessment of Intimacy in Relationships instrument, which has been validated in cancer patients [37, 38] and the Experiences in Closer Relationship Scale-Short Form [39]. Both patients and partners will rate how helpful and upsetting they perceive each other to be during times when they needed support.

### Sample size

We will randomize 75 dyads to the CBMB intervention, AC, and WLC groups (25 dyads per group).

### Recruitment

Research staff identifies potential participants via MD Anderson’s computerized appointment system for the thoracic clinics. We approach potential participants during clinic visits or infusion appointments, screen them for eligibility, and ask them for consent. If a patient’s partner is not present during the initial contact, we ask the patient for permission to contact the partner via telephone to obtain consent.

### Randomization

Couples are assigned to one of the three study groups using minimization randomization [40]. Factors used for randomization include the prognostic factors of age, sex, ECOG performance status score [41–43], and distress score according to the National Comprehensive Cancer Network (NCCN) distress thermometer [44]. The NCCN distress thermometer is a 10-point visual analog scale with anchors of “no distress” to “extreme distress.”

### Blinding

This is an unmasked trial. However, research staff involved in data collection is blind to group allocation.

### Data collection

Dyads in all three groups are assessed at three time points: baseline, 4 weeks later, and 3 months later.

### Statistical methods

To evaluate our study aims of this ongoing trial and determine whether the CBMB intervention should be further evaluated in a subsequent larger trial, we will follow the steps described below.

#### Specific aim 1

The primary objective of this research is to determine feasibility according to overall accrual, attrition, completion of questionnaires, and session adherence. We will calculate rates, frequencies, and 90% confidence intervals when applicable and judge the trial to be feasible if at least 30% of eligible couples consent to participate (i.e., approach 250 couples to accrue 75). Moreover, we will deem the trial feasible if at least 70% of enrolled couples (≥ 54) are retained and complete the 4 weeks and 3 months follow-up assessments (i.e., complete ≥ 80% of the entire assessment to be counted as a completer) and at least 50% of all CBMB and AC sessions are attended.

#### Specific aim 2

We will perform descriptive analyses of QOL in patients and their partners, including calculations of means, standard deviations, and confidence intervals.

As an *exploratory aim*, we will explore descriptive analyses including cross-sectional and prospective correlations of measures of mindfulness, compassion, holding back, and intimacy and indices of QOL at each time point, to help provide the basis for an underlying mechanism of the intervention to be further explored in a future larger trial. Additionally, we will calculate the intraclass coefficients for patient and partner variables to further provide a rationale for this dyadic intervention.

#### Sample size considerations

Assuming a rather conservative 30% attrition rate, a *post-attrition* sample size of 54 patients or partners (18 per group) will yield 80% power to detect a difference in mean pre-post treatment FACT-SP change of 2.31 between CBMB and AC groups and between the CBMB and WLC groups, each assuming a SD of pre-post change of 2.54 (based on our pilot data) [11]. However, this trial is not designed to examine intervention efficacy, and we have not carried out any formal power calculations. In line with recommendations in the literature, we will focus on examining feasibility and descriptive analyses [45–47]. We selected the sample size of 75 dyads based on previously published psycho-oncological feasibility trials, in which the investigators typically enrolled 20–25 participants/dyads per study group [48].

## Discussion

This study will provide the necessary evidence of the feasibility of an RCT of a CBMB intervention for couples coping with mNSCLC, a vulnerable yet understudied population, using videoconference delivery. Given the paucity of existing RCTs of interventions for behavioral supportive care interventions for patients with advanced cancer, findings of this research will be of interest to other researchers working in this area or wanting to examine recruitment for other technology-based intervention studies in similar populations.

We acknowledge that the proposed study sample excludes nonromantic family caregivers (e.g., adult children) and patients with other forms of aggressive lung cancer, such as extensive small-cell lung cancer. Given that this is a small trial, we believe that a rather homogenous sample is justifiable at this stage of development. However, if our feasibility criteria are not met, we will expand the trial to other family caregivers and types of lung cancer. Once the trial’s feasibility is established to inform a future larger study, we will design that RCT with a sample size that will be informed by clinically meaningful changes in study outcomes. We will present findings of this feasibility trial at national and international behavioral medicine and supportive care conferences and submit them for publication in peer-reviewed journals.

### Trial status

Participant recruitment started on March 15, 2017 and is expected to be finished by July 2018.

## Abbreviations

AC: Active control
CBMB: Couple-based mind-body
ECOG: Eastern Cooperative Oncology Group
FACT-SP: Functional Assessment of Cancer Therapy - Spiritual Well-Being Scale
mNSCLC: Metastatic non-small cell lung cancer
NCCN: National Comprehensive Cancer Network
QOL: Quality of life
RCT: Randomized controlled trial
WLC: Wait list control
